# Effects of Glucocorticoid Therapy on Sepsis Depend Both on the Dose of Steroids and on the Severity and Phase of the Animal Sepsis Model

**DOI:** 10.3390/life12030421

**Published:** 2022-03-14

**Authors:** Ye Jin Park, Min Ji Lee, Jinkun Bae, Jung Ho Lee, Han A Reum Lee, Sehwan Mun, Yun-seok Kim, Chang June Yune, Tae Nyoung Chung, Kyuseok Kim

**Affiliations:** 1 Department of Emergency Medicine, CHA University School of Medicine, Seongnam 13497, Gyeonggi, Korea; yejin6577@naver.com (Y.J.P.); minji.lee29@gmail.com (M.J.L.); galen97@chamc.co.kr (J.B.); quiet93@outlook.kr (J.H.L.); harlee91@naver.com (H.A.R.L.); bogmi0415@gmail.com (S.M.); loupys@naver.com (Y.-s.K.); june1976@hanmail.net (C.J.Y.); hendrix74@cha.ac.kr (T.N.C.); 2 Department of Emergency Medicine, CHA Bundang Medical Center, CHA University, Seongnam 13497, Gyeonggi, Korea

**Keywords:** sepsis, glucocorticoid, immunosuppression, cytokines, rats

## Abstract

Steroids are currently being used in sepsis, particularly in septic shock. However, clinical trials to date have shown contradictory results. This could be attributed to the different patient endotypes and steroid doses, which have also contributed to the inconclusive results. We investigated the effects of glucocorticoid therapy on sepsis in a polymicrobial sepsis model in a variety of settings, such as steroid dose, severity, and sepsis phase. We used a rat model of fecal slurry polymicrobial sepsis. First, we investigated the optimum dose of steroids in a sepsis model. We administered different doses of dexamethasone after sepsis induction (0.1DEX; 0.1 mg/kg, 0.2DEX; 0.2 mg/kg, 5DEX; 5 mg/kg). Second, we used two different severities of the fecal slurry polymicrobial sepsis rat model to examine the effects of the steroids. A moderate or severe model was defined as a survival rate of approximately 70% and 30%, respectively. Third, we administered steroids in an early (1 h after sepsis induction) or late phase (25 h after sepsis). In all the experiments, we investigated the survival rates. In the determined optimal model and settings, we measured serum lactate, alanine transferase (ALT), creatinine, tumor necrosis factor-α (TNF-α), interleukin (IL)-6, IL-10, and arterial blood gas. We evaluated the bacterial burden in the blood and spleen. Endotoxin tolerance of peripheral blood mononuclear cells (PBMCs) and splenocytes was also investigated to determine the level of immune suppression 24 h after sepsis by measuring TNF-α production after stimulation with lipopolysaccharide (LPS) in an ex vivo model. Early treatment of 0.2 mg/kg dexamethasone in a severe sepsis model showed the best beneficial effects. In moderate- or late-phase sepsis, there was no survival gain with steroid treatment. DEX0.2 group showed less acute kidney injury manifested by serum creatinine and blood urea nitrogen. DEX decreased the levels of cytokines, including IL-6, IL-10, and TNF-α. Colony-forming units were significantly decreased in the blood when administered with dexamethasone. Endotoxin tolerance was not significantly different between the DEX0.2 and control groups. In conclusion, early treatment of 0.2 mg/kg dexamethasone improved the outcomes of rats in a severe sepsis model.

## 1. Introduction

Sepsis is defined as a life-threatening organ dysfunction caused by dysregulated host responses to infection [1]. Worldwide, it has a high incidence and mortality and has become a public health problem [2,3,4]. Given this background, the World Health Organization has announced that sepsis is a global health priority [5]. Many drugs have been developed for the treatment of sepsis, but none have shown clinical efficacy.

The current guideline recommends low-dose steroids in refractory septic shock; however, the evidence is weak [6].

Studies on the effects of steroids in sepsis have shown different settings in terms of steroid dose, sepsis severity manifested by the mortality rate of the control group, and timing of steroid administration. Steroids might be beneficial in patients with a higher baseline mortality rate [7,8] compared to the group with a relatively lower mortality rate. Concomitantly, considering the immunosuppressant nature of steroids, it might be harmful when administered in the immunosuppressant phase of sepsis, that is, usually in the late phase of sepsis. Therefore, it may be more effective for early treatment.

Historically, high-dose steroids have been used; however, there have been no ultimate survival gains [9,10,11]. Subsequently, low-dose steroids have been advocated, but in recent years, large and well-designed clinical trials have shown contradictory results [8,12], leaving questions about the usefulness of steroids in septic shock. Some meta-analyses advocate for different doses of steroids (200–400 mg hydrocortisone per day) with current guidelines of 200 mg in sepsis [13]. In addition, there was a small randomized controlled trial with a slightly higher dosage of steroids (dexamethasone 0.2 mg/kg) in sepsis than in the guidelines, but they lacked a group with a routine dose of steroids [14].

With this background, we hypothesized that the effect of steroids on sepsis might differ according to steroid dosage, severity, and phase of sepsis. To investigate this, we used a polymicrobial intra-abdominal infection (IAI) model in rats.

## 2. Materials and Methods

### 2.1. In Vivo Sepsis Model Induction

This study was approved by the Institutional Animal Care and Use Committee of our institute, in accordance with the National Institutes of Health Guidelines (IACUC-210043). Experiments were performed on male Sprague-Dawley rats (body weight, 270–330 g) purchased form the Orient Bio Inc. (Seongnam, Korea). The rats were housed in a controlled environment (room temperature 20~24 °C, humidity 40~60%) in a specific pathogen-free room with 10 and 14 h of light and dark exposure, respectively. Animals had standard food and water ad libitum for 14 days before the experiment.

We used a body weight-adjusted polymicrobial IAI sepsis model according to our previous study [15,16]. In brief, donor rats were anesthetized with an intramuscular injection of Zoletil (50 mg/kg) and xylazine (10 mg/kg). A midline laparotomy was performed, and the cecum was extruded. A 0.5 cm incision was made in the antimesenteric surface of the cecum, and the cecum was squeezed to expel feces. The feces were collected weighed, and it was diluted with 5% dextrose saline at a ratio of 1:3. In sepsis induction, rats were anesthetized as above, and a 0.5 cm midline laparotomy was performed, and fecal slurry was administered into the peritoneal cavity. The amount of fecal slurry was decided according to the severity of sepsis. In this study, 6.5 and 4.5 mL/kg fecal slurry were administered to induce severe and moderate sepsis, respectively. The fecal slurry was vortexed to obtain a homogeneous suspension before administration into the intraperitoneal cavity. The volume of fecal slurry administered to each animal was adjusted based on the body weight of the recipient rat. We administered subcutaneous fluid resuscitation (30 mL/kg 5% dextrose saline), and imipenem was injected subcutaneously at a dose of 25 mg/kg twice daily for 2 days. Thereafter, the stratified randomization by weight was performed by assistant of the experiments.

**Experiment 1**: Survival study for optimum dose of steroids in a severe septic shock model.

The mortality rate of the control group in this severe septic shock model was approximately 70%. In this study, we administered dexamethasone at different doses via the tail vein 1 h after induction of sepsis. Rats were randomly allocated to the following groups: dexamethasone 0.1 mg/kg (DEX0.1), 0.2 mg/kg (DEX0.2), 5 mg/kg (DEX5), and vehicle (control (Con)) groups. Survival was monitored every 12 h for 14 days.

**Experiment 2**: Survival study in the moderate septic shock model.

We tested the effects of steroids in a moderate septic shock model with a target mortality rate of 30% in the control group. Different dexamethasone doses were administered as described above.

**Experiment 3**: Survival study according to the timing of steroid administration.

In this study, we compared the effects of dexamethasone timing (early vs. late). The early DEX0.2 group received dexamethasone 1 h after sepsis induction. On the contrary, the late group received dexamethasone 25 h after the sepsis induction.

**Experiment 4**: Endotoxin tolerance study: Ex vivo PBMC and splenocyte stimulation with LPS.

In this study, we used a severe sepsis model. There were three groups: sepsis only (control group), sepsis with early 0.2 mg/kg dexamethasone (DEX0.2), and no sepsis (sham). PBMCs and splenocytes were isolated at each time point after sepsis induction. PBMCs were isolated using a Ficoll gradient method, as previously described [17]. Isolated PBMCs were stimulated with LPS to observe and compare the levels of immune paralysis. TNF-α levels were measured 5 h after LPS stimulation. Briefly, isolated PBMCs were seeded at a density of 1 × 10^5^ cells/mL in 96-well plates, and 100 ng/mL LPS (Escherichia coli O111: B4; Sigma-Aldrich, St. Louis, MO, USA) was added to each well. After 5 h, the culture medium was collected, and TNF-α levels were analyzed using a TNF-α enzyme-linked immunoassay (ELISA) kit (ab236712, Abcam, Cambridge, MA, USA).

Splenocytes were isolated and stimulated with lipopolysaccharide (LPS) to compare immune paralysis. TNF-α levels were measured 5 h after LPS stimulation. Briefly, isolated splenocytes were seeded at a density of 5 × 10^5^ cells/mL in 6-well plates, and 1 μg/mL LPS (Escherichia coli O111: B4; Sigma-Aldrich, St. Louis, MO, USA) was added to each well. After 5 h, the culture medium was collected, and TNF-α was analyzed using a TNF-α ELISA kit (R&D Systems Inc., Minneapolis, MN, USA).

**Experiment 5**: Tissue study: In this study, blood and tissues were harvested 24 h after sepsis induction. We used a severe sepsis model. There were three groups: sepsis only (control group), sepsis with early 0.2 mg/kg dexamethasone (DEX0.2), and no sepsis (sham). The colony-forming units (CFUs) in the blood and spleen were counted. We measured plasma chemistries, arterial blood gases, and cytokines.

In all experiments, sepsis was verified by observing reduced motor activity, lethargy, shivering, and piloerection [18]. We did not think confounders could affect the results with this study design; thus, it was not controlled.

### 2.2. Colony-Forming Unit (CFU) Assay

Blood and spleen samples were used to count bacterial CFUs in sepsis-induced rats. A total of 30 mg of spleen tissue was homogenized in 500 μL of DPBS and centrifuged at 12,000 rpm for 10 min at 4 °C. The cell pellets were resuspended in 1 mL of DPBS to form the stock samples. The stock samples were diluted 1:10 with DPBS. To count blood CFUs, 800 μL of DPBS was added to 200 μL of blood. The samples were spread on TSA agar plates (BD Biosciences) without antibiotics and incubated at 37 °C overnight, and the bacterial colonies were counted for analysis. A CFU study was performed in a severe and moderate sepsis model.

### 2.3. Plasma Chemistry Assay: ALT, Albumin, Blood Urea Nitrogen (BUN), Creatinine, and Lactate Dehydrogenase

Serum alanine aminotransferase, albumin, BUN, creatinine, and lactate dehydrogenase levels were measured using a Chemistry Analyzer (AU480, Beckman Coulter, Brea, CA, USA).

### 2.4. Arterial Blood Gas Analysis

Arterial blood gas analysis was performed using arterial blood samples via the abdominal artery (ABL90 Flex Plus, Radiometer, Copenhagen, Denmark). Blood sample was collected using a heparinized syringe.

### 2.5. Cytokine Measurements

The levels of the cytokines IL-6 (R6000B, R&D Systems, MN, USA), IL-10 (ab214566, Abcam, MA, USA), and TNF-α (ab236712, Abcam, Cambridge, MA, USA) in plasma were measured using ELISA kits according to the manufacturer’s instructions. The optical density at 450 nm was measured using a microplate reader (VersaMax with SoftMax Pro software, Molecular Devices, San Jose, CA, USA).

## 3. Statistical Analysis

The Shapiro-Wilk test was performed to determine the normality of the data. Normally distributed data are presented as mean ± standard deviation and were compared using independent *t* tests. If the data did not fit a normal distribution, they were presented as the median and interquartile range and were analyzed using the Mann-Whitney U test or Kruskal-Wallis test. Fisher’s exact test was used for categorical variables. Survival rates were compared using the Kaplan-Meier log-rank test. Statistical significance was defined as a *p* value of <0.05. All analyses were performed using easy R software.

## 4. Results

### 4.1. Survival Study According to the Dose of Steroid Depending on Severity of Sepsis

**Figure 1:**
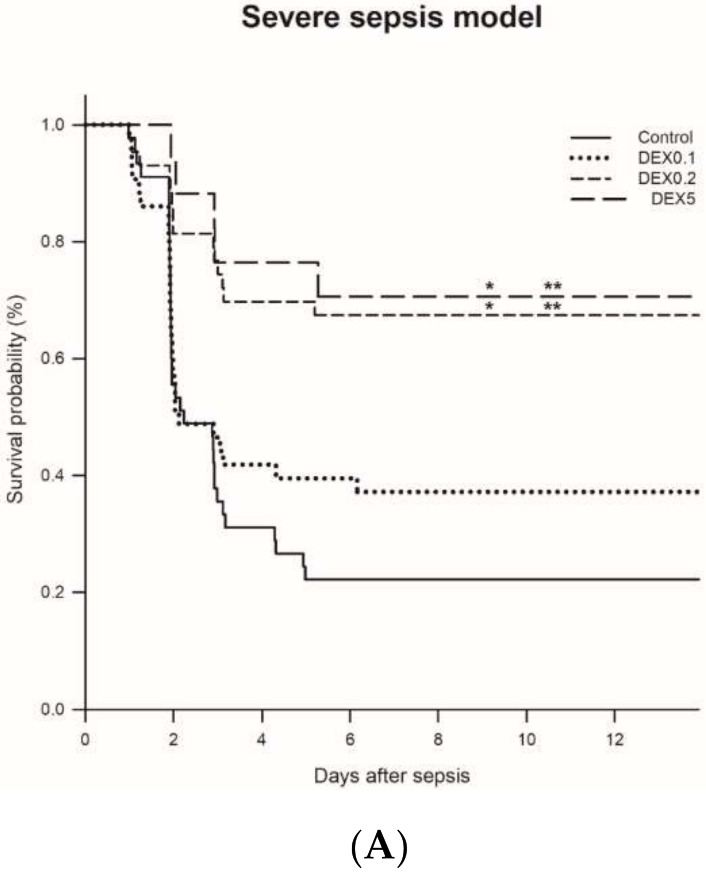

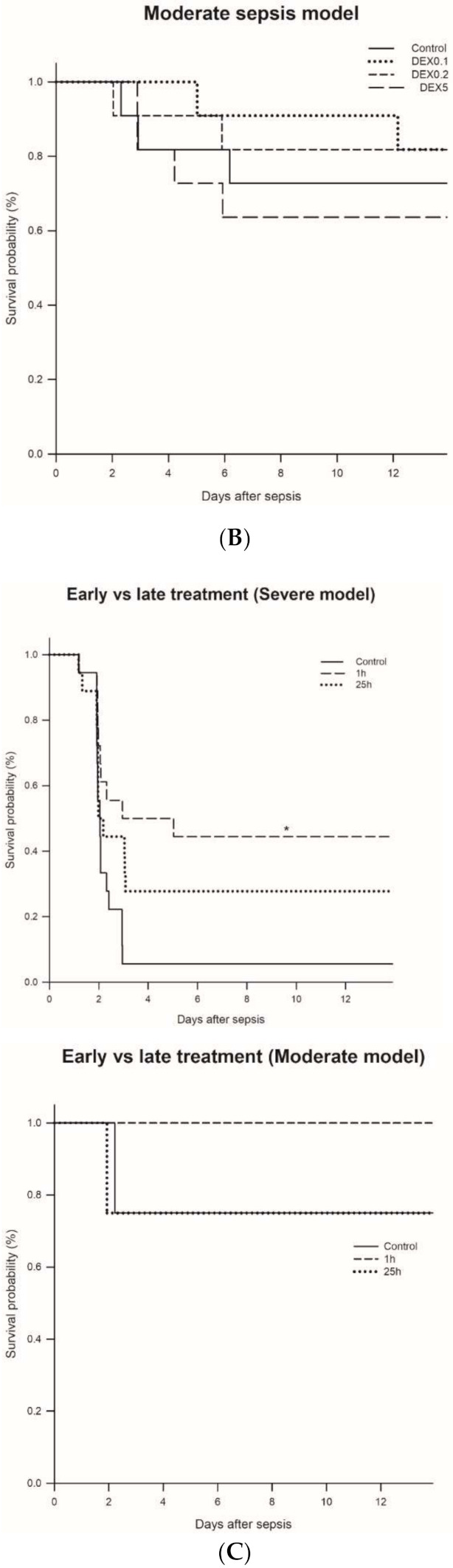
Survival effects of dexamethasone on the polymicrobial sepsis model according to the different doses of dexamethasone, severity of sepsis, and phase of sepsis. (**A**) In a severe sepsis model. (Drug was administered 1 h after sepsis. Control and DEX0.1, DEX0.2, *n* = 43 to 45 per group, DEX5 *n* = 17). * *p* < 0.01 compared with control; ** *p* < 0.05 compared with DEX0.1. (**B**) In a moderate sepsis model. (Drug was administered 1 h after sepsis, *n* = 11 per group). (**C**) In early vs. late phase sepsis. (Severe sepsis model was used in upper panel. *n* = 18 per group. Moderate sepsis model in lower panel. *n* = 4 per group). * *p* < 0.05 compared with control.

In the severe sepsis model, DEX0.2 and DEX5 showed increased survival rates compared to the control and DEX0.1 group (Figure 1A).

In the moderate sepsis model, there were no survival differences between the control, DEX0.1, DEX0.2, and DEX5 groups; however, there was a decreased survival tendency in the DEX5 group (Figure 1B).

### 4.2. Survival Study According to The Phase of Sepsis

Early administration of dexamethasone (1 h after sepsis induction) increased the survival rate of sepsis. The delayed use of steroids (25 h after sepsis induction), however, was not effective in sepsis in terms of survival rate (Figure 1C).

### 4.3. Serum Creatinine, BUN, and ALT

**Figure 2:**
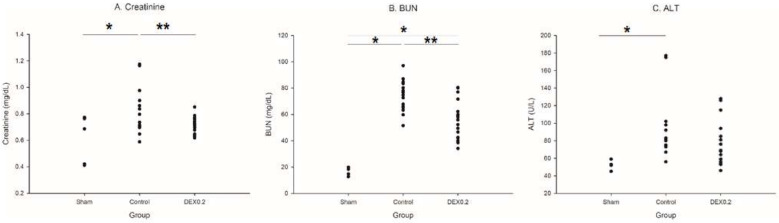
Effects of dexamethasone on organ injury in polymicrobial severe sepsis model. (**A**) Serum creatinine. (Sham *n* = 6; control and DEX0.2, *n* = 15 to 17 per group). ** *p* < 0.05 compared with control using Mann-Whitney. (**B**) Serum BUN. (Sham *n* = 4; control and DEX0.2, *n* = 15 to 18 per group). * *p* < 0.01 compared with sham; ** *p* < 0.05 compared with control using ANOVA. (**C**) Serum ALT. (Sham *n* = 4; control and DEX0.2, *n* = 15 to 17 per group). * *p* < 0.01 compared with sham using Kruskal-Wallis.

Serum creatinine and BUN were significantly decreased in the DEX0.2 group (Figure 2). Serum ALT levels did not differ between the groups.

### 4.4. Cytokines

**Figure 3:**
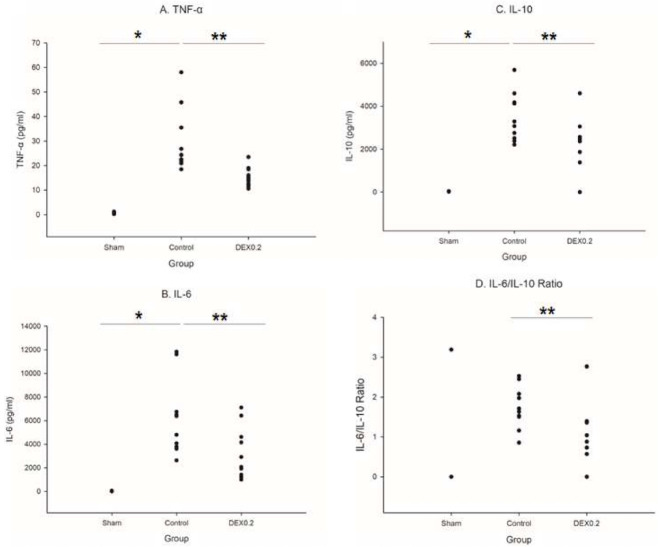
Effects of dexamethasone on inflammation in a polymicrobial severe sepsis model. (**A**) Plasma TNF-alpha. (Sham *n* = 4; Control and DEX0.2, *n* = 10 to 11 per group). * *p* < 0.05 compared with sham; ** *p* < 0.05 compared with control using Kruskal-Wallis. (**B**) Plasma IL-6. (**C**) Plasma IL-10. (**D**) Plasma IL-6/IL-10 ratio. (Sham *n* = 4; Control and DEX0.2, *n* = 10 per group). * *p* < 0.05 compared with sham using Kruskal-Wallis; ** *p* < 0.05 compared with control using Mann-Whitney or *t*-test.

Plasma TNF-α, IL-6, and IL-10 levels were significantly lower in the DEX0.2 group than in the control group (Figure 3).

### 4.5. CFU Assay

**Figure 4:**
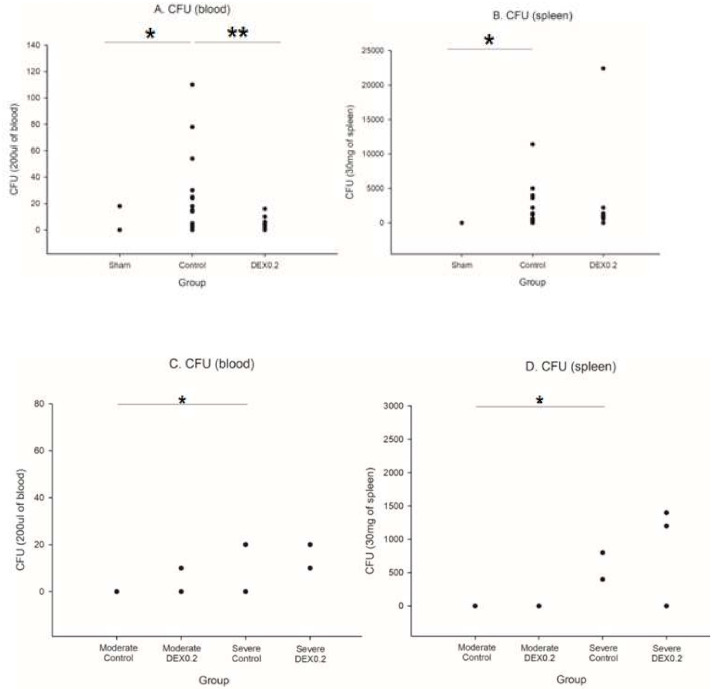
CFU in the blood and spleen in a polymicrobial sepsis model. (**A**) Blood CFU in severe model. (**B**) Spleen CFU in severe model. (Sham *n* = 6; control and DEX0.2, *n* = 15 per group). (**C**) Blood CFU in moderate and severe model. (**D**) Spleen CFU in moderate and severe model (*n* = 2–3 per group). * *p* < 0.05 compared with sham using Kruskal-Wallis; ** *p* < 0.05 compared with control using Mann-Whitney. CFU, colony-forming units.

In the severe sepsis model, the CFU in blood was significantly decreased in the DEX0.2 group than in the control group. However, CFU in the spleen was not different between the two groups (Figure 4). There was no significant difference in CFU in the moderate sepsis model.

### 4.6. Endotoxin Tolerance (Ex Vivo PBMC and Splenocyte Stimulation with LPS)

**Figure 5:**
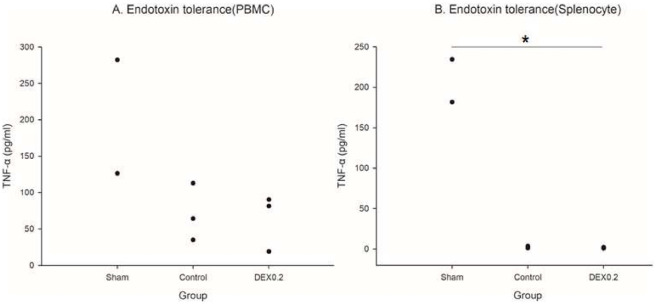
Effects of dexamethasone on endotoxin tolerance in a polymicrobial severe sepsis model. (**A**) PBMC. (**B**) Splenocyte. (*n* = 2 to 6 per group). * *p* < 0.05 compared with sham using Kruskal-Wallis. PBMC, peripheral blood mononuclear cell; LPS, lipopolysaccharide.

In both splenocytes and PBMCs, there were no significant differences in endotoxin tolerance between the control and DEX0.2 group (Figure 5).

### 4.7. Other Laboratory Findings

**Figure 6:**
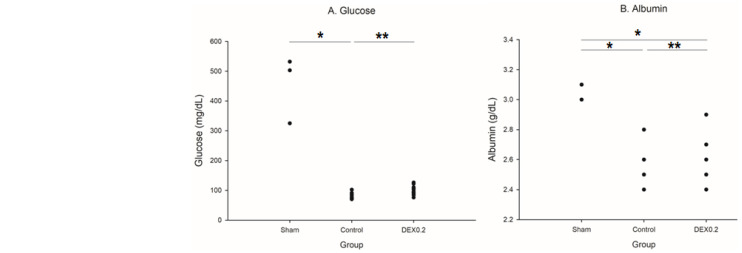

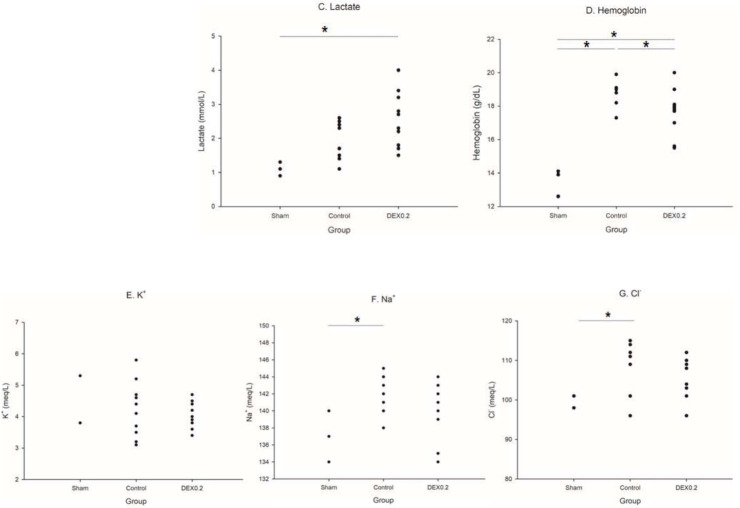
Findings of various laboratories in a polymicrobial severe sepsis model. (**A**) Serum glucose, (**B**) serum albumin, (**C**) plasma lactate, (**D**) hemoglobin, (**E**) serum potassium, (**F**) serum sodium, (**G**) serum chloride. (Sham *n* = 3; control and DEX0.2, *n* = 9 to 11 per group). * *p* < 0.05 compared with sham or control using Kruskal-Wallis or ANOVA; ** *p* <0.01 compared with control using *t*-test.

Serum glucose levels were significantly increased in the DEX0.2 groups. Serum albumin was significantly decreased in sepsis, which was attenuated by dexamethasone. Other chemical results were not different between the groups. Hemoglobin and hematocrit were significantly elevated in the control group (Figure 6).

### 4.8. Arterial Blood Gas Analysis and Lactate

**Figure 7:**
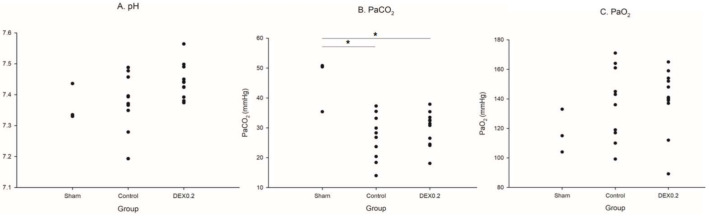
Arterial blood gas analysis in a polymicrobial severe sepsis model. (**A**) pH, (**B**) PaCO^2^, (**C**) PaO^2^. (Sham *n* = 3; control and DEX0.2, *n* = 10 to 11 per group). * *p* < 0.05 compared with sham using ANOVA.

There were no significant differences in the arterial blood gas analysis results, but a tendency for a decreased pH in the control group was observed (Figure 7).

## 5. Discussion

This study is the first to demonstrate the effects of steroids in sepsis in various settings with regard to steroid dose, severity, and phase of sepsis. Early treatment with 0.2 mg/kg dexamethasone in the severe sepsis model showed the best effect.

Steroid treatment increased the survival rate in the severe sepsis model only, with a mortality rate of approximately 70%, but not in the 30% model. This is partly in line with the results of previous clinical trials. The effects of steroids have been reported in clinical trials. It is usually effective in severe septic shock, with high mortality rates [7,8], but ineffective in less severe patients [12,19,20]. With this background, surviving sepsis campaign guidelines recommend steroids for severe septic shock [6,21]. Previous animal studies have reported contradictory results regarding the effects of steroids on sepsis according to the severity of sepsis [22,23]. However, even in studies with the conclusion that hydrocortisone was effective regardless of the severity of sepsis, there were statistical difference in survival according to severity [22].

We chose the dose of dexamethasone based on a literature review. The dose of steroids has been extensively studied. Historically, high doses of steroids have been investigated since the 1970s, but there has been no survival gain [9,10,11]. At that time, methylprednisolone 30 mg/kg was administered, which could be 5–6 mg/kg of dexamethasone. In the early 2000s, low doses of steroids were used in clinical trials, and some reported beneficial effects [1,7,8,12,19]. The guidelines recommend 200 mg/day of intravenous hydrocortisone. However, this did not provide strong evidence, with weak strength and of low quality [6]. Some meta-analyses reported that 200–400 mg per day of hydrocortisone or equivalent was most likely to benefit septic patients [13]. This dose could be converted to 8–16 mg of dexamethasone, and in 80 kg body weight patients, it could be 0.1–0.2 mg/kg. There was one small randomized controlled trial that reduced the mortality rate in septic shock with the usage of 0.2 mg/kg of dexamethasone [14]. In COVID-19, 6 or 12 mg/day of dexamethasone is useful for clinical outcomes [24]. With all these data, we chose 0.1, 0.2, and 5 mg/kg of dexamethasone in this study, and we found that 0.2mg/kg could be more beneficial.

We studied the effects of steroids on the sepsis phase. Traditionally, initial hyperinflammatory phase of sepsis has been known to occur, followed by compensatory immune suppression [25]. Recent concepts have changed, and both pro-inflammatory and anti-inflammatory reactions might occur concomitantly [26]. Even in this theory, the early use of an anti-inflammatory agent could be more beneficial since pro-inflammatory reaction starts early in sepsis. Dexamethasone has anti-inflammatory effects; thus, it might have more beneficial effects if used in the early hyperinflammatory phase, which is consistent with the results of this study. Our study showed that inflammatory cytokines were attenuated by early administration of dexamethasone. Moreover, in sepsis, especially in the late phase, immunosuppression is well known to occur, and this has been reported to be associated with mortality [27,28,29,30]. Steroids are well-known immune suppressants, and we hypothesized that steroid treatment in the later phase of sepsis could be ineffective or even harmful with respect to immune function. In this study, not late, but early administration of dexamethasone showed survival gain, supporting our hypothesis. The results of this study were not consistent with those of a previous study [22], which showed no difference in survival in terms of the timing of steroid treatment. The difference between our study and previous studies is the timing of steroid use. We used 24 h after sepsis as a late treatment, versus a previous study that used 12 h.

Sepsis induces acute kidney injury [31]. In this study, the early use of dexamethasone in severe sepsis mitigated acute kidney injury, which was shown by a lower serum creatinine level and BUN.

The major concerns in the use of steroids in sepsis are infectious complications such as secondary infection. We investigated CFU in the blood and spleen, and the administration of dexamethasone did not increase bacterial burden. On the contrary, bacterial burden was reduced in blood in the DEX group. We also tested endotoxin tolerance in PBMC and the spleen. Endotoxin tolerance has been proposed as one of the mechanisms of immunosuppression in sepsis [32]. In this study, we found that the administration of dexamethasone did not affect endotoxin tolerance. With these data, we might infer that 0.2 mg/kg DEX did not cause infectious complications.

Lactate levels were not different between the steroid and control groups. It is well known that the administration of steroids can increase lactate [2,33,34], which is known as type B hyperlactatemia. Sepsis induces hyperlactatemia (type A), and we believe that dexamethasone reduced type A hyperlactatemia, but increased type B hyperlactatemia, and this resulted in no difference in lactate levels compared to the control group.

Initial hemoconcentration in sepsis is related to significant hypovolemia, and it is related to the severity of sepsis [35]. In this study, hemoconcentration was observed, and treatment with dexamethasone significantly attenuated hemoconcentration.

The results of this pre-clinical study with the previous literature [13] could be applied to clinical trials in the field of steroids in sepsis. First, the dose of steroids should be tested beyond 200 mg/day of hydrocortisone. Our results suggest that a 400 mg/kg dose may be more effective. Second, continuous infusion of steroids should be challenged. The current guidelines regarding the use of steroids in septic shock do not comment on the exact method of administering hydrocortisone. Moreover, previous guidelines recommend continuous infusion of hydrocortisone. Considering that septic shock is life-threatening and that the recommended timing of steroid therapy is during refractory shock to fluid and vasopressors, a more rapid action of steroids is mandatory. However, the continuous infusion of steroids could fall short of this expectation. If we use continuous infusion in compliance with the guidelines, the total dose of hydrocortisone administered in the first hour is less than 10 mg, which is an absolutely small dose of steroid in life-threatening, rapidly deteriorating septic shock. In line with this, the intermittent administration of hydrocortisone was better in shock reversal than continuous infusion in a recent clinical study [36]. In this respect, a bolus injection of steroids could be considered. Third, the timing of steroid treatment should be considered. This may be related to the severity of septic shock. In this study, steroid treatment was effective for early and severe sepsis. This finding suggests that steroid treatment might be started early, for example, not in the intensive care unit (ICU), but in the emergency department in subsequent clinical trials, especially in severe septic shock. Currently, most studies regarding steroid treatment in sepsis enrolled patients in the ICU.

Considering the complexity of the effects of steroids on sepsis, investigated here, future clinical trials on the use of steroids might be performed to narrow target patients, for example, early vs. late phase, severity of sepsis, and/or different dose/duration of steroid use.

This study has several limitations. First, we used dexamethasone rather than hydrocortisone or methylprednisolone, which are commonly used in sepsis. Dexamethasone is longer acting, and in animal experiments where repeated doses could be limited, it might be more useful. In addition, a recent study on COVID-19 used dexamethasone [24]. Second, we could not translate this finding into a real clinical situation. Although fecal slurry or cecal ligation and puncture models seem to be the best models for mimicking clinical sepsis, there are gaps between preclinical and clinical sepsis. Therefore, we are performing clinical trials using this concept (NCT05136560). Third, sepsis has various pathophysiologies, and this could be different according to the sepsis model; thus, another sepsis model could be used to test the concept of this study. Fourth, we did not specify the mechanisms underlying these findings. Although we investigated plasma cytokine levels and endotoxin tolerance, it is not enough to determine the mechanism of action. This requires further investigation. Fifth, we could not investigate the side effects of the steroids. However, hyperglycemia and hypernatremia were not evident in this study. Although some adverse effects would have occurred in this model, it would not seriously affect mortality, since mortality rates were lower in each steroid group compared to the control group.

In conclusion, early treatment of 0.2 mg/kg dexamethasone improved the outcomes in a severe polymicrobial sepsis model. These results can be tested in clinical trials for sepsis.

## Data Availability

The datasets generated and analyzed during the current study are available from the corresponding author on reasonable request.
